# Editorial: Case reports in gastrointestinal cancers : 2022

**DOI:** 10.3389/fonc.2023.1333174

**Published:** 2023-11-16

**Authors:** Pashtoon Murtaza Kasi

**Affiliations:** Department of Oncology/Hematology, Weill Cornell Medicine, New York, NY, United States

**Keywords:** precision oncology, precision medicine, N-of-1 clinical trials, case reports, editorial, CtDNA, exceptional responders, N-of-1 analysis

With the focus of journals and conferences on clinical trials and large registries, the value of unique individual cases cannot be underestimated. These individual case stories that tell compelling presentations and outcomes can be helpful.

The opportunity to oversee these submissions to the Research Topic within gastrointestinal cancers was of interest to me since it brings to focus a diverse array of case reports. Often, these are what the National Cancer Institute (NCI) calls the “exceptional responders” (1). It brings to light these patients with unique cancers and exceptional clinical outcomes. **Figure 1** illustrates how each patient from diverse backgrounds and different cancers with their treatment journey typifies an individual story, and it needs to be viewed as such.

**Figure 1:**
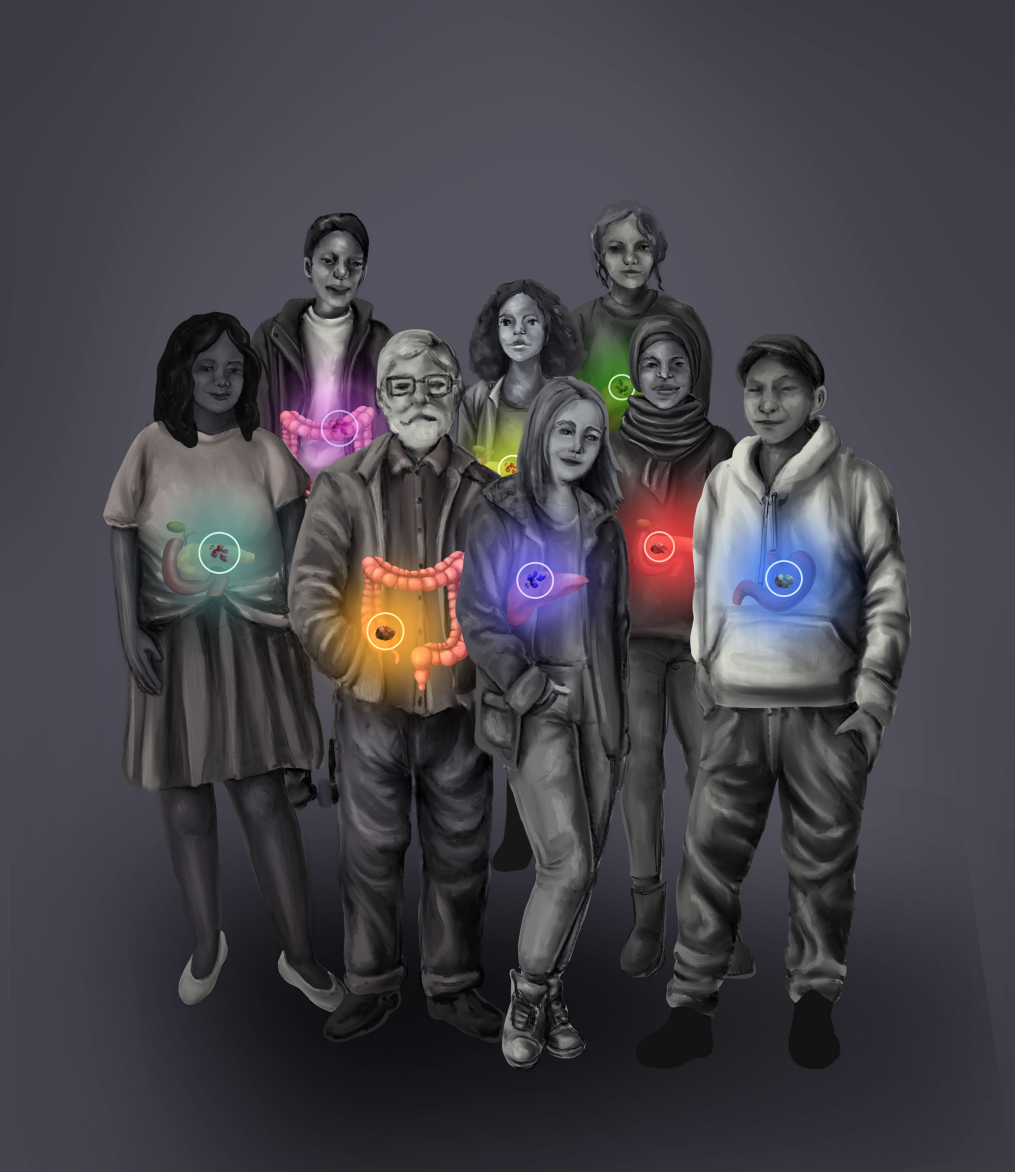
Illustration showing how each patient from diverse backgrounds and different cancers with their treatment journey typifies an individual story.

If we look at the spectrum of these individual reports, some are deep, brisk, durable, and sometimes curative outcomes to different kinds of novel therapies. Some are new or off-label usage of drugs for actionable markers in histologies or cancer types or settings for which they still are not approved by regulatory agencies or guidelines. Not all are about exceptional outcomes. Some of these reports highlight unexpected or not previously known side effects of drugs or point to mechanisms and/or ways to overcome and manage these issues. These can help inform the care of future patients. Not only that, from a practical standpoint, publications in peer-reviewed journals can help provide evidence supporting a strategy that biologically makes sense but needs clinical reports of similar stories. Insurance companies and guidelines committees seek more evidence before recommending or endorsing an approach for access to off-label therapies.

Advances are coming in subsets for patients with different types of cancers. We are also seeing agnostic approvals for markers like fusions that are rare events but very actionable. Real-world evidence and publications in this regard can be clinically meaningful. We have also found these helpful as initial proof-of-principle published evidence for investigator-initiated trials that can change practice.

Lastly, from an academic standpoint, these are also opportunities for students, clinical investigators, and multidisciplinary teams to have a place or home to highlight their work. I want to laud that the journal was open to case reports and hope readers, scientists, patients, caregivers, advocacy groups, and physicians who would benefit from this endeavor. Future directions are changes in clinical trial designs proposing n-of-1 trials as an opportunity to help expedite drug development (2).

## Author contributions

PK: Writing – original draft, Writing – review & editing.
